# Supplementary material for: The current global perspective of the knowledge-attitude-behavior of the general public towards the corona virus disease -19 pandemic: Systematic review and meta-analysis on 67,143 participants
Source: PLoS One. 2021 Dec 17;16(12):e0260240. doi: 10.1371/journal.pone.0260240 (PMC8682882; doi:10.1371/journal.pone.0260240)
Supplement: S1 Table — (DOCX) [file pone.0260240.s001.docx]

|  | “Coronavirus” OR “corona” OR “COVID” OR “COVID-19” OR “COVID19” OR “COVID-2019” OR “severe acute respiratory syndrome” OR “SARS-COV-2” |
| --- | --- |
| AND | “knowledge” OR “attitude” OR “practice” OR “awareness” OR “perception” OR “belief” OR “feeling” OR “worrisome” OR “fear” OR “anxiety” OR “prevention” OR “behavior” OR “cross section” OR “survey” OR “questionnaire” OR “observation” OR “community” OR “public” OR “population” OR “participants” |

A sample of search strategy in Medline database using MeSH keywords:
