# Supplementary material for: The current global perspective of the knowledge-attitude-behavior of the general public towards the corona virus disease -19 pandemic: Systematic review and meta-analysis on 67,143 participants
Source: PLoS One. 2021 Dec 17;16(12):e0260240. doi: 10.1371/journal.pone.0260240 (PMC8682882; doi:10.1371/journal.pone.0260240)
Supplement: Attachment — Submitted filename: Response to reviewers-revised2.docx [file pone.0260240.s005.docx]

**Reviewers' Comments** **to the Authors:**

**Reviewer #3: I have scanned the authors' work meticulously. The authors have tried to react to certain questions; nevertheless, publication bias and quality evidence assessment issues has not been addressed yet. Hint : Publication bias can be assessed objectively using statistical tests like Beggs test, Egger test and harbord test using Stata version 16/ its extension or comprehensive meta analysis software whereas quality evidence assessment can be made using Grade pro soft ware. The authors are suggested to down load the aforementioned softwares and use it.**

**Authors’ Response:**

We have performed quality evidence assessment of each reported outcome and used Grade pro software for that as suggested. In addition, both Begg’s and Egger’s tests were used to evaluate symmetry of funnel plots for assessment of publication bias. We have reported all the findings in the revised manuscript. We have utilized MedCalc software for meta-analysis per authors’ convenience. Thank you for your valuable suggestion.
