# Supplementary material for: The current global perspective of the knowledge-attitude-behavior of the general public towards the corona virus disease -19 pandemic: Systematic review and meta-analysis on 67,143 participants
Source: PLoS One. 2021 Dec 17;16(12):e0260240. doi: 10.1371/journal.pone.0260240 (PMC8682882; doi:10.1371/journal.pone.0260240)
Supplement: Attachment — Submitted filename: Response to reviewers-revised3.docx [file pone.0260240.s006.docx]

**Reviewers' Comments** **to the Authors:**

**Reviewer #3: I have meticulously scanned the authors manuscript. My concerns were addressed. Nevertheless, there was some concerns. eg in abstract : background : the authors should state his purpose by using the aim instead of using goal, Materials and methods in main text: spelling error should be corrected: review manager not review manage**

**Authors’ Response:**

As per the third reviewer’s suggestion, we have replaced the word “goal” with “aim” in the abstract and we have corrected the spelling of “review manager” in the methods section. Thank you for your valuable feedback.
