# Supplementary material for: The current global perspective of the knowledge-attitude-behavior of the general public towards the corona virus disease -19 pandemic: Systematic review and meta-analysis on 67,143 participants
Source: PLoS One. 2021 Dec 17;16(12):e0260240. doi: 10.1371/journal.pone.0260240 (PMC8682882; doi:10.1371/journal.pone.0260240)
Supplement: Attachment — Submitted filename: Response to reviewers-revised4.docx [file pone.0260240.s007.docx]

**Reviewers' Comments** **to the Authors:**

**Reviewer #3: The overall prevalence of knowledge, positive attitude, worrisome, and practice of precautionary measures should be indicated along with 95% confidence interval both in the abstract and main body part of the results.**

**Authors’ Response:**

As per the third reviewer’s suggestion, we ensured that all pooled prevalence are associated with 95% confidence interval in both abstract and results sections. Thank you for your comment.
