# Supplementary material for: The current global perspective of the knowledge-attitude-behavior of the general public towards the corona virus disease -19 pandemic: Systematic review and meta-analysis on 67,143 participants
Source: PLoS One. 2021 Dec 17;16(12):e0260240. doi: 10.1371/journal.pone.0260240 (PMC8682882; doi:10.1371/journal.pone.0260240)
Supplement: Attachment — Submitted filename: Response to reviewers-revised6.docx [file pone.0260240.s008.docx]

**Comments of Editor:**

**Editor:** **Thank you for revising the draft. This manuscript has been substantially improved. However, few changes are suggested by the reviewer. Moreover, It is advised to take assistance from native English speaker to revise the draft for Grammar, Syntax and spelling.**

**Authors’ Response:**

We have initially consulted with a professional medical writing service that corrected the mechanical errors and performed linguistic editing in the manuscript. The manuscript was revised again and grammatical errors were corrected.

**Reviewers' Comments** **to the Authors:**

**Reviewer #3: Abstract**

**Background**

**There was tense error eg**

**The aim of this review is to form a preliminary and contemporary understanding of the general public knowledge, attitude, and behaviors towards the COVID-19 pandemic global**

**Authors’ Response:**

The manuscript was revised for any tense error and corrected accordingly. All changes have been implemented in the final manuscript. Thanks for your suggestion.

**Reviewer #3:**

**Results**

**The overall prevalence of knowledge, positive attitude, worrisome, and practice of precautionary measures can't be computed using RevMan software. Use other POWERFUL software like STATA or other software.**

**Authors’ Response:**

We have calculated pooled prevalence of studies using RevMan Software which computes the prevalence as the effect size along with 95% confidence interval. Since RevMan has an option of calculating pooled prevalence, we decided to stick with this software. Thank you for the suggestion.
